# Genetic evidence for a potential causal relationship between insomnia symptoms and suicidal behavior: a Mendelian randomization study

**DOI:** 10.1038/s41386-022-01319-z

**Published:** 2022-05-10

**Authors:** Malik Nassan, Iyas Daghlas, John W. Winkelman, Hassan S. Dashti, International Suicide Genetics Consortium, Richa Saxena

**Affiliations:** 1 Division of Sleep Medicine, Beth Israel Deaconess Medical Center, Harvard Medical School, Boston, MA USA; 2 Center for Genomic Medicine, Massachusetts General Hospital, Harvard Medical School, Boston, MA USA; 3 Harvard Medical School, Boston, MA USA; 4 Departments of Psychiatry and Neurology, Massachusetts General Hospital, Harvard Medical School, Boston, MA USA; 5 Center for Genomic Medicine and Department of Anesthesia, Critical Care and Pain Medicine, Massachusetts General Hospital, Harvard Medical School, Boston, MA USA; 6 Program in Medical and Population Genetics, Broad Institute, Cambridge, MA USA; 7 Present Address: Mesulam Center for Cognitive Neurology and Alzheimer’s Disease, Northwestern University, Chicago, IL USA; 8 Present Address: Department of Neurology, University of California, San Francisco, San Francisco, CA USA

**Keywords:** Genetic markers, Risk factors, Psychiatric disorders

## Abstract

Insomnia and restless leg syndrome (RLS) are associated with increased risk for suicidal behavior (SB), which is often comorbid with mood or thought disorders; however, it is unclear whether these relationships are causal. We performed a two-sample Mendelian randomization study using summary-level genetic associations with insomnia symptoms and RLS against the outcomes of risk of major depressive disorder (MDD), bipolar disorder (BP), schizophrenia (SCZ), and SB. The inverse-variance weighted method was used in the main analysis. We performed replication and sensitivity analyses to examine the robustness of the results. We identified outcome cohorts for MDD (*n* = 170,756 cases/329,443 controls), BP (*n* = 20,352/31,358), SCZ (*n* = 69,369/236,642), SB-Cohort-2019 (*n* = 6569/14,996 all with MDD, BP or SCZ; and SB within individual disease categories), and SB-Cohort-2020 (*n* = 29,782/519,961). Genetically proxied liability to insomnia symptoms significantly associated with increased risk of MDD (odds ratio (OR) = 1.23, 95% confidence interval (CI) = 1.2–1.26, *P* = 1.37 × 10^–61^), BP (OR = 1.15, 95% CI = 1.07–1.23, *P* = 5.11 × 10^–5^), SB-Cohort-2019 (OR = 1.17, 95% CI = 1.07–1.27, *P* = 2.30 × 10^–4^), SB-Cohort-2019 in depressed patients (OR = 1.34, 95% CI = 1.16–1.54, *P* = 5.97 × 10^–5^), and SB-Cohort-2020 (OR = 1.24, 95% CI = 1.18–1.3, *P* = 1.47 × 10^–18^). Genetically proxied liability to RLS did not significantly influence the risk of any of the outcomes (all corrected *P* > 0.05). Results were replicated for insomnia with MDD and SB in Mass General Brigham Biobank and were consistent in multiple lines of sensitivity analyses. In conclusion, human genetic evidence supports for the first time a potentially independent and causal effect of insomnia on SB and encourages further clinical investigation of treatment of insomnia for prevention or treatment of SB.

## Introduction

Insomnia and restless leg syndrome (RLS) have emerged as modifiable risk factors for mood disorders and suicidal behavior (SB) [1–3]. Insomnia is a clinical diagnosis characterized by difficulty falling or staying asleep that is associated with distress and/or dysfunction [4, 5]. Insomnia disorder has a prevalence of 10–20.0% [4, 6]. Cross-sectional and longitudinal observational studies demonstrate that insomnia is associated with increased risk for psychiatric disorders [1]. Sleep disturbances have been associated with an increased longitudinal risk for bipolar disorders (BP) ((odds ratio (OR) = 1.72)) and depressive disorders (OR = 1.62) [7]. More specifically, in a meta-analysis of prospective cohort studies, insomnia was associated with an increased risk of depression (pooled relative risk was 2.27) [8]. Furthermore, in a more recent meta-analysis, insomnia was associated with increased longitudinal risk for SB [suicidal ideation OR = 2.10, suicide attempts OR = 1.78, suicide deaths OR = 1.54] [9]. However, more research is needed to uncover whether these associations represent causal relationships and mechanisms underlying the connection between insomnia and suicide. Notably, schizophrenia (SCZ) has a known association with insomnia, mood disorders, and SB that is not fully characterized [10–13]. Insomnia is heritable with heritability estimated from twin studies to be 0.39 [14]. This has motivated genome-wide association studies, which have identified over 200 SNPs associated with insomnia symptoms [15].

RLS is a clinical diagnosis in which predominantly nighttime leg restlessness at rest, relieved by movement, leads to distress and sleep disturbance. The prevalence of clinically significant RLS is estimated to be 2.5% [16]. Cross-sectional and longitudinal observational studies demonstrate that RLS is associated with an increased risk for MDD, reduced quality of life, and overall increased mortality [17–24]. RLS is also associated with an increased risk of suicide and self-harm (adjusted hazard ratio was 2.66) [2]. Twin studies have estimated RLS heritability to be up to 70%, with a 19.6% SNP-based heritability [25, 26]. This establishment of heritability paved the way for genome-wide association studies (GWAS) that have identified 20 SNPs that are significantly associated with RLS [26].

Most studies testing the relationships between insomnia, RLS and mood disorders and SB are observational studies. Observational studies (even longitudinal cohorts) can establish associations between risk factors and diseases, but they are insufficient to establish causal relationships [27, 28]. Mendelian randomization (MR) is an analytic method that uses genetic proxies of exposures to test associations with disease outcomes, and has several distinct advantages for causal inference [29, 30]. Recent large GWAS for insomnia, RLS, mood disorders, and most recently SB, now permit the use of MR to investigate causal relationships between these associated disorders and behaviors. Of note, SB is a complex behavior and caused by genetic and environmental factors; with estimated heritability from twin studies of 30–55% [31, 32].

In this study, we aimed to utilize MR to assess for the first time whether genetically proxied insomnia symptoms and RLS have causal relationships with SB, and to examine this relationship in the presence of mood and thought disorders.

## Methods

### Ethical approval and patient consent

Deidentified summary statistics and publicly available data were utilized in this study, and thus no IRB approval was required for the analyses.

### Study design

**Fig. 1:**
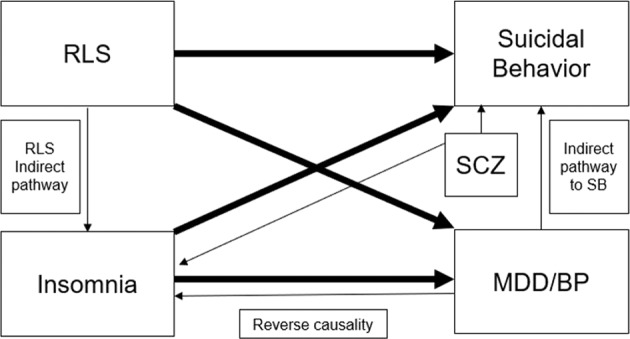
The hypothesized relationships between the studied exposures and outcomes. Bold arrows represent the direct effect between exposures and outcomes. Thin arrows represent indirect pathways. BP bipolar disorder, SB suicidal behavior, RLS restless leg syndrome, SCZ schizophrenia, MDD major depressive disorder.

In this study, we applied a two-sample MR study design using summary-level genetic association data [33]. As exposures, we used previously identified genetic variants for insomnia symptoms and RLS to test for their potential causal effects on mood disorders (MDD and BP), SCZ, and SB. Our main and sensitivity MR analyses were structured to demonstrate the following assumptions: (1) genetic variants are robustly associated with the studied exposures (e.g., insomnia symptoms and RLS), (2) associations of the genetic variants with the exposures and with the outcomes are not confounded, and (3) the genetic variants are influencing the risk of the outcomes through the exposures, and not through alternative pathways. The relationships between the studied exposures and outcomes are illustrated in [Fig. 1].

### Genetic associations with the exposures

We identified genetic proxies for liability to insomnia symptoms (as surrogate for insomnia) as genome-wide significant variants from the largest published insomnia GWAS at the time of analysis [Table 1] [34]. This GWAS meta-analysis included insomnia cases of European ancestry from the UK Biobank (UKB: cases 109,402/controls 277,131) and 23andMe cohorts (cases 288,557/controls 655,920). In the UKB: insomnia complaints were evaluated by asking: “Do you have trouble falling asleep at night or do you wake up in the middle of the night?” Insomnia cases were defined as participants who answered this question with “usually”, while participants answering “never/rarely” or “sometimes” were defined as controls. For 23andMe, insomnia cases were confirmed by a positive response to at least one of these questions: “Have you ever been diagnosed with, or treated for: Insomnia?”; “Have you ever been diagnosed with, or treated for, any of the following conditions: Insomnia but not Narcolepsy, Sleep apnea or Restless leg syndrome”; “Has a doctor ever told you that you have any of these conditions: Insomnia (difficulty getting to sleep or staying asleep)?”; “Have you ever been diagnosed by a doctor with any of the following neurological conditions: Sleep disturbance”; “Do you routinely have trouble getting to sleep at night?”; “What sleep disorders have you been diagnosed with? Please select all that apply: Insomnia, trouble falling or staying asleep”; “Have you ever taken these medications? Prescription sleep aids”; “In the last 2 years, have you taken any of these medications? Prescription sleep aids”. Insomnia definition had higher accuracy in the UKB cohort than in the 23andMe cohort when compared to the diagnostic cut-off of Insomnia Severity Index and Pittsburgh Sleep Quality Index in an independent sample from the Netherlands Sleep Registry (sensitivity/specificity: UKB = 98/96%; 23andMe = 84/80%). Of note, both scales have high reliability and validity in diagnosing insomnia [35, 36]. Furthermore, in the same independent sample, the UKB insomnia questions had good sensitivity of 94% and specificity of 89% in differentiating cases and controls in comparison with structured interview [37]. A total of 250 independent genetic variants (low pairwise linkage disequilibrium (*r*^2^ < 0.1)) were associated with insomnia in the meta-analysis of the UKB and 23andMe cohorts at genome-wide significance (*P* < 5 × 10^–8^) [Supplementary Table 1S].

**Table 1:** Summary of the GWAS cohorts included in the analyses.

Study	Disease	Cases	Controls	Number of genome-wide SNPs
Jansen et al. *Nat Genet* (2019)	Insomnia	397,959	933,051	250
Schormair et al. *Lancet Neurol* (2017)	RLS	45,896	382,638	20
Howard et al. *Nat Neurosci* (2019)	MDD	170,756	329,443	102
Stahl et al. *Nat Genet* (2019)	BP	20,352	31,358	30
Ripke et al. *MedRxiv* (2020)	SCZ	69,369	236,642	270
Mullins et al. *American Journal of Psychiatry* (2019)	SB 2019	6569	14,996	0
Mullins et al. *Biol Psychiatry* (2021)	SB 2020	29,782	519,961	2

We selected genome-wide genetic association data from the largest published meta-analysis of RLS GWAS to generate a genetic proxy for RLS [Table 1] [26]. This study included three cohorts in the meta-analysis (EU-RLS GENE, INTERVAL consortia, and 23andMe) in which all participants were of European ancestry. RLS diagnosis varied across the cohorts and included diagnosis by face-to-face interview by an expert neurologist for EU-RLS GENE, through the validated Cambridge-Hopkins Restless Legs Questionnaire for the INTERVAL consortia, and through a single question survey for the 23andMe cohort “Have you ever been diagnosed with restless legs syndrome?”. The collective discovery and replication samples consisted of 45,896 cases and 382,638 controls. Twenty independent genetic variants (low pairwise linkage disequilibrium (*r*^2^ < 0.01)) were associated with RLS at genome-wide significance (*P* < 5 × 10^–8^) [Supplementary Table 2S]. All GWAS were analyzed with standard quality control procedures, including methods to control for population stratification.

### Genetic associations with the outcomes

We identified publicly available GWAS summary statistics for the psychiatric outcomes of interest (MDD, BP, SCZ, and SB). To limit confounding by ancestral differences, we selected studies limited to individuals of European ancestry. These studies are listed in [Table 1]. Beta coefficients of the genetic variants associated with the exposure and outcome phenotypes were harmonized by matching effect alleles.

Summary statistics GWAS were obtained from the Psychiatric Genomics Consortium (PGC) online database for MDD, BP, SCZ [https://www.med.unc.edu/pgc/download-results/]. We included two GWAS for SB: (1) SB-Cohort-2019, available from PGC and included cases with either MDD, BP, or SCZ; and provided subset cohorts for analyses stratified by disease, (2) SB-Cohort-2020 which is the largest GWAS for SB, and included the previous SB-Cohort-2019.

For MDD, the most recent meta-GWAS included a total of 246,363 cases and 561,190 controls; we had access to summary statistics from combined data set from UKB and PGC (*n* = 170,756 cases/329,443 controls) and not from 23andMe [38]. The UKB MDD diagnosis was based on self-reported help-seeking for “problems with nerves, anxiety, tension or depression” (termed ‘broad depression’), while the PGC cohort utilized a range of depression phenotypes (including structured clinical interview as well as broader criteria). A total of 102 genome-wide independent variants were associated with MDD in the meta-analysis.

For BP, the most recent meta-GWAS included a total of 20,352 cases and 31,358 controls of European descent (collected from 32 studies); and replication analysis of 822 variants (*P* < 1 × 10^−4^) in 9412 cases and 137,760 controls [39]. The combined analysis identified 30 genome-wide significant genetic variants influencing the risk of BP. Overall, cases needed to meet DSM-IV, International Classification of Diseases (ICD)-9, or ICD-10 criteria for a lifetime diagnosis of BP (by either structured diagnostic instrument, clinician-administered checklists, or medical record review). Most controls were examined for the absence of any other lifetime psychiatric disorders. A total of 30 genome-wide independent variants were associated with MDD in the meta-analysis.

For SCZ, the most recent meta-GWAS included a total of 69,369 cases and 236,642 controls [40]. This was the largest combined cohort yet from PGC (included 90 cohorts) and identified 270 independent genome-wide significant genetic loci. Of note, this study included 80% of the sample from European ancestry and 20% from East Asian ancestry. Cases included diagnoses of SCZ or schizoaffective disorder; details of each of the enrolled cohorts are available in the manuscript [40]. A total of 270 genome-wide independent variants were associated with MDD in the meta-analysis.

Two cohorts were used for the outcome of SB (encompassing here both fatal and non-fatal suicidal attempts). The first cohort is a PGC cohort (SB-Cohort-2019) published in 2019 (cases 6569, controls 14,996) and stratified by comorbid psychiatric disorder (MDD, BP, or SCZ) [41]. The subjects were obtained from 16 MDD cohorts, 21 BP cohorts, and 9 SCZ cohorts from PGC, where data on suicide attempts had been gathered. Only patients affected by the three psychiatric disorders were included and all three psychiatric disorders were defined using structured psychiatric interviews. All individuals were of European ancestry. Items from structured clinical interviews offered data on suicidal attempts. Lifetime suicidal attempt was characterized across cohorts as an intentional act of self-harm with the intent to result in death. Individuals who only endorsed suicidal ideation were not included as cases. Across the cohorts, there was a sum of 6569 individuals who attempted suicide and 17,232 individuals who had not attempted suicide. No genome-wide significant associations were identified in the meta-analysis.

The second cohort (SB-Cohort-2020) assessed for SB in 29,782 cases and 519,961 controls and is the most recent and largest SB GWAS [pre-print released in 2020, and publication was in 2021] [42, 43]. The cohorts included samples from European ancestry (the majority of the cases), admixed African American ancestry (4%), and East Asian ancestry (6%). This GWAS included 21 cohorts, of which cases were individuals who died by suicide (2 cohorts) or made a non-fatal suicide attempt (19 cohorts) which is defined as a lifetime act of intentional self-harm with intent to cause one’s own death. Individuals who only endorsed suicidal ideation were not included as cases. Information on suicidal attempts was obtained via structured clinical interviews for 15 cohorts, self-report questionnaires for 2 cohorts, and ICD codes or hospital records for 2 cohorts. Cases of death by suicide (2 cohorts) were obtained from the Medical Examiner’s Office of the Hyogo Prefecture and the Division of Legal Medicine, at the Kobe University Graduate School of Medicine in Japan or the Utah State Office of the Medical Examiner. A percentage of cases from the Columbia University and iPSYCH cohorts included individuals with death by suicide that was established through the Columbia Classification Algorithm for Suicide Assessment and the Cause of Death Register in Denmark, respectively. Two genome-wide significant genetic variants were identified as influencing the risk of SB.

### Mendelian randomization analyses

All analyses were performed in R Version 3.5.3 using the TwoSampleMR v0.4.229 package. The inverse-variance weighted (IVW) method was the main MR method used to estimate the effect of genetically proxied liability to insomnia symptoms or RLS on each of the psychiatric outcomes [44]. The corrected statistical significance threshold is *P* less than 3.13 × 10^–3^, accounting for 16 statistical comparisons across 2 exposures and 8 outcomes.

We then performed multiple sensitivity analyses to assess the robustness of the findings. First, we removed SNPs in LD between the insomnia symptoms and RLS genetic proxies to reduce any confounding between these exposures (removing SNPs within 10 Mb and with *r*^2^ ≥ 0.7). Second, we removed SNPs from the exposure genetic proxies (i.e., insomnia symptoms and RLS) that were in LD (using a more stringent threshold *r*^2^ ≥ 0.01) with an outcome that was significant in the main MR IVW analysis (MDD, BP, and SB-Cohort-2020). Third, we performed model-based sensitivity analyses that relax various MR assumptions regarding pleiotropy, including: MR-egger regression, weighted median, and MR-PRESSO [44, 45]. We also created leave-one-out plots to display the results from the IVW and Egger regression analyses, to assess for outliers.

We sought to replicate our findings in the Mass General Brigham (MGB) Biobank (formerly Partners Biobank) for the outcomes of MDD, BP, and SB only (MDD *n* = 4640/23,849, BP *n* = 145/28,344, SB *n* = 1054/27,435) [46]. The MGB Biobank is a hospital-based cohort study from the MGB healthcare network in Boston, MA with electronic health record (EHR) and genetic data. Recruitment for the Biobank launched in 2010 and remains ongoing at participating clinics and electronically. Recruitment strategy has been described previously [47]. All recruited patients provided consent written informed upon enrollment. The present study protocol was approved by the MGB Institutional Review Board (#2018P002276). Effect estimates for MDD, BP, and SB were generated using data for 30,683 participants with genetic data and limited to participants of European ancestry [48]. Cases of MDD, BP, and SB were determined from EHR using a validated algorithm based on natural language processing of structured and unstructured data including coded diagnoses, medications, procedures, and vital signs [46]. The remaining participants were set as control. To determine SNP effects on MDD, BP, and SB, we performed genetic association analysis in unrelated participants of European ancestry with PLINK logistic regression and an additive genetic model adjusted for age, sex, five principal components, and genotyping array [49].

Finally, we ran a reverse MR IVW analysis between MDD and BP (using the genome-wide genetic proxies from the same GWAS) as exposures and insomnia symptoms as outcome (from the same insomnia symptoms GWAS but only including the UKB cohort due to lack of public availability of 23andMe data).

## Results

### Main analyses

**Fig. 2:**
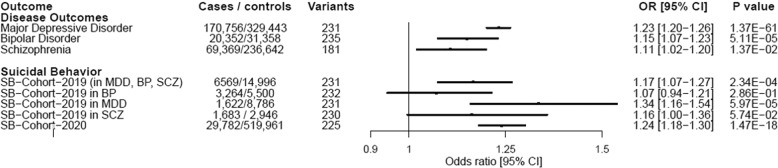
Forest plot of inverse-variance weighted MR effects of genetically proxied liability to insomnia symptoms on psychiatric outcomes. Boxes reflect point estimates and surrounding lines reflect 95% confidence intervals. CI confidence interval, OR odds ratio, BP bipolar disorder, MDD major depressive disorder, RLS restless leg syndrome, SB suicidal behavior, SCZ schizophrenia.

**Fig. 3:**
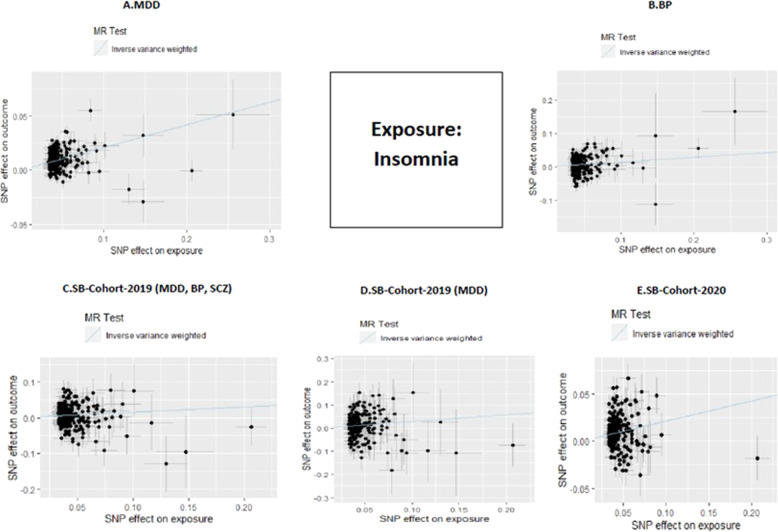
Mendelian randomization scatterplots for effects of genetic liability to insomnia on psychiatric outcomes. BP bipolar disorder, MDD major depressive disorder, SB suicidal behavior, SCZ schizophrenia.

**Fig. 4:**
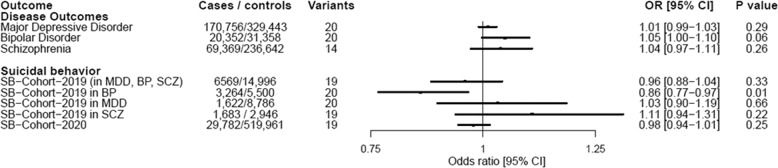
Forest plot of inverse-variance weighted MR effects of genetically proxied liability to RLS on psychiatric outcomes. Boxes reflect point estimates and surrounding lines reflect 95% confidence intervals. CI confidence interval, OR odds ratio, BP bipolar disorder, MDD major depressive disorder, RLS restless leg syndrome, SB suicidal behavior, SCZ schizophrenia.

MR analyses showed significant associations of genetically proxied insomnia symptoms with MDD, BP and SB [MDD (OR = 1.23, 95% CI = 1.2–1.26, *P* = 1.37 × 10^–61^), BP (OR = 1.15, 95% CI = 1.07–1.23, *P* = 5.11 × 10^–5^), SB-Cohort-2019 (OR = 1.17, 95% CI = 1.07–1.27, *P* = 2.30 × 10^–4^), and SB-Cohort-2020 (OR = 1.24, 95% CI = 1.18–1.3, *P* = 1.47 × 10^–18^)] [Fig. 2]. Analyses for the outcome of SB in the 2019 cohort stratified by disease status showed that the effect of insomnia symptoms on SB is most robust in the depressed population (OR = 1.34, 95% CI = 1.16–1.54, *P* = 5.97 × 10^–5^) [Fig. 2]. The scatterplots for the significant findings are included in Fig. 3 and Supplementary Table 3S. On the other hand, genetically proxied liability to RLS was not associated with any of the study outcomes [Fig. 4 and Supplementary Table 4S].

### Sensitivity analyses

Results for the effects of genetic liability to insomnia symptoms on the outcomes were unchanged when we removed SNPs in LD between insomnia symptoms and RLS genetic proxies, and when we removed SNPs in LD between insomnia symptoms and the significant outcomes (MDD, BP, and SB-cohort-2020) [Supplementary Tables 5S–9S].

Model-based MR sensitivity analyses were performed to assess the robustness of effects on potential horizontal pleiotropy [Table 2]. Results from the weighted median and MR-PRESSO were overall similar to results from the IVW analysis. However, the MR-Egger regression for the effect of genetically proxied liability to insomnia symptoms on SB had non-significant findings, and a point estimate in the opposite direction. We performed a leave-one-out MR-Egger analysis to assess whether the results were driven by an outlier [Supplementary Figs. S1–S5]. This showed that a single outlier SNP (rs113851554 in *MEIS1*) was driving the effect in the opposite direction for MDD, SB in MDD, and SB-Cohort-2020. Notably, this *MEIS1* SNP is also a strong risk factor for RLS and may reflect heterogeneity in the insomnia discovery GWAS based on undiagnosed RLS or pleiotropy at this locus [50]. For BP another single outlier SNP (rs9527083 intergenic in chromosome 13) was driving the effect in the opposite direction. For SB-Cohort-2019 no single outlier was driving in the opposite direction. For comparison, we ran leave-one-out analyses for the IVW method and found no clear outliers [Supplementary Figs. S6–S10].

**Table 2:** MR main and sensitivity analyses results.

Exposure	Outcome	Outcomes *N* case/cont	Sensitivity analyses	*N* SNPs^a^	OR	95% CI		*P*
Insomnia	MDD	170,756/329,443	IVW	231	1.23	1.20	1.26	**1.37** **×** **10**^**–61**^
			egger_regression	231	1.09	0.99	1.20	0.07
			weighted_median	231	1.18	1.15	1.21	**3.49** **×** **10**^**–35**^
			Presso^b^	na	1.23	1.20	1.26	**8.75** **×** **10**^**–44**^
Insomnia	BP	20,352/31,358	IVW	235	1.15	1.07	1.23	**5.11** **×** **10**^**–5**^
			egger_regression	235	1.33	1.02	1.73	**0.03**
			weighted_median	235	1.13	1.04	1.22	**2.39** **×** **10**^**–3**^
			Presso^b^	na	1.16	1.09	1.24	**6.33** **×** **10**^**–6**^
Insomnia	SB-cohort-2019	6569/14,996	IVW	231	1.17	1.07	1.27	**0.0002**
			egger_regression	231	0.85	0.61	1.18	0.34
			egger (removing *MEIS1* rs113851554)	230	0.86	0.59	1.26	0.44
			weighted_median	231	1.13	1.01	1.26	**0.04**
			Presso	na	na	na	na	na
Insomnia	SB in MDD	1622/8786	IVW	231	1.34	1.16	1.54	**5.97** **×** **10**^**–5**^
			egger_regression	231	1.10	0.62	1.93	0.75
			weighted_median	231	1.28	1.04	1.58	**0.018**
			Presso	na	na	na	na	na
Insomnia	SB-cohort-2020	29,782/519,961	IVW	225	1.24	1.18	1.30	**1.47** **×** **10**^**–18**^
			egger_regression	225	0.99	0.81	1.21	0.9
			egger (removing *MEIS1* rs113851554)	224	1.04	0.82	1.31	0.74
			Weighted_median	225	1.18	1.12	1.25	**4.61** **×** **10**^**–10**^
			Presso^b^	na	1.23	1.18	1.28	**4.02** **×** **10**^**–18**^

^a^Exposure SNPs available in the outcome.

^b^Presso outliers: MDD (“rs113851554” “rs2431108” “rs2815757” “rs55772859” “rs73163783”), BP (rs2431108 “rs324017” “rs4090240” “rs521484” “rs6973090”), SB-cohort-2020 (“rs10502966” “rs12666306” “rs1937447” “rs56133505” “rs73671843”).

Bolded *P* values indicate significant association.

We sought to replicate the findings in the MGB Biobank, an independent clinical biobank, using available data for MDD, BP and SB. These analyses showed replication of the findings for the effect of genetic liability to insomnia symptoms on MDD (OR = 1.12, 95% CI = 1.03–1.21, *P* = 0.0096) and SB (OR = 1.19, 95% CI = 1.01–1.40, *P* = 0.03) but not for BP (*P* = 0.58). The MGB cohort had a small BP case sample size (*n* = 145) and consequently large confidence intervals in the analysis, but the effect was in the same direction (OR = 1.14) [Supplementary Table 10S]. Finally, the reverse MR IVW analysis between MDD and BP as exposures and insomnia symptoms as outcome showed that genetic liability to MDD but not BP is a risk factor for insomnia symptoms [Supplementary Table 11S].

## Discussion

In this two-sample MR study we found for the first-time evidence for a potentially independent and causal effect of insomnia symptoms on SB, and further strengthened the evidence for insomnia being a potential causal risk factor for MDD and BP. However, genetically proxied RLS (which can be comorbid with insomnia) was not associated with any tested psychiatric outcomes, indicating that our findings for insomnia were not driven by RLS. These results were replicated, and consistent across several lines of sensitivity analyses. This is the first comprehensive study analyzing the causality of insomnia symptoms and RLS for SB utilizing MR.

We found a robust association between insomnia symptoms and MDD (OR = 1.23, *P* = 1.7 × 10^–61^), which is consistent with the existing literature [34]. In addition, when we sub-classified SB by disorder, the most robust association of insomnia symptoms with SB was within the MDD sub-group. However, LD analyses, sensitivity MR analyses, and a replication analysis in an independent sample all demonstrated insomnia as an independent risk factor for SB independently of MDD. Our results are in line with a recent prospective study in which insomnia was an independent risk factor for SB, with an effect that was more pronounced among patients with MDD (of the total effect, 32% was mediated by MDD) [51].

Identifying insomnia symptoms as a potentially causal risk factor SB is important from both mechanistic and clinical perspectives. One proposed “stress accumulation” hypothesis is that insomnia is associated with the insufficient dissolution of emotional distress (due to REM sleep dysfunction and fragmentation) which can theoretically lead to emotional distress accumulation. On the other hand, an insomnia effect on the frontal cortex might disrupt emotion regulation and lead to disinhibition [52]. Collectively, insomnia-induced stress accumulation and behavioral disinhibition might be part of how insomnia causes SB. Another proposed hypothesis implicates the hyperarousal state found in insomnia that has been conceptualized as a biomarker for suicide. This hypothesis implicates agitation, irritability, and hypervigilance as mediators between insomnia and SB [53, 54].

Our finding that insomnia is a causal risk factor for MDD and SB further supports the treatment of insomnia in patients with MDD and SB. Treatment of insomnia and depression can be done concomitantly or sequentially [55, 56]. Our finding of a bidirectional effect (since reverse MR showed MDD increased the risk for insomnia symptoms) supports a concurrent treatment approach for both disorders. Our results also support the investigation of treatment of insomnia for the prevention of SB. A recent clinical trial utilizing the sedative zolpidem—although not meeting the primary outcome of reducing the scores on the Scale for Suicide Ideation—demonstrated a reduction in suicidal ideation on the Columbia-Suicide Severity Rating Scale in depressed patients with more severe insomnia [57]. On the other hand, an observational study found that cognitive behavioral therapy for insomnia (CBT-I) was associated with a 65% reduction in OR of suicidal thoughts, independent of changes in depression [58]. However, large randomized controlled trials evaluating sleep medications and CBT-I effects on SB are still warranted [59, 60].

The association of sleep disturbance with BP is well established [61]. Furthermore, treating insomnia in BP patients (via CBT-I) improved sleep, mood, and functioning [62]. The use of sedative hypnotics to treat mania is a common practice although more clinical trials are needed [63]. Although not passing Bonferroni level of statistical significance, insomnia symptoms were associated with a nominal increase in SCZ risk. This is in line with their known comorbidity and reported increased severity of SCZ and worsening clinical outcomes due to insomnia [11].

Genetically proxied RLS was not significantly associated with any of the studied outcomes. This could be due to multiple reasons. First, the previous observational studies might have not adjusted for relevant variables that were driving the effect (like other sleep disturbance for example). One study found that the RLS-depression association might be partially explained by sleep disturbance and periodic limb movements [21]. Second, the phenotyping of RLS in previous studies might have created a heterogeneous group of patients (such as using a survey question vs a clinical diagnosis by an expert sub-specialist). The poor diagnostic performance of survey instruments for RLS has recently been established [64]. Lastly, larger GWAS in more homogeneous phenotype samples may produce a more accurate genetic proxy of RLS that can be utilized to re-test these associations in the future.

The key strength of this study is the use of MR, which reduces bias due to confounding and assesses causality rather than association. Furthermore, the use of large sample sizes (by selecting the largest published GWAS) leads to more precise estimates of MR effect sizes. This study did not only look at SB in general, but also looked at SB classified by diagnosis which is a rigorous approach. Another strength is that we were able to replicate our findings for insomnia as a risk factor for MDD and SB in an independent sample from MGB biobank.

This study has limitations to consider. Although sensitivity analyses (including MR-PRESSO) were consistent with the main results, horizontal pleiotropy cannot be completely excluded [65]. On the other hand, the discovery samples for the variants associated with insomnia asked questions more consistent with insomnia symptoms rather than an insomnia disorder diagnosis, although as mentioned above those insomnia symptoms had a good correlation with validated scales. Furthermore, as UK Biobank and 23andMe participants are healthier than the general population, our findings may not be generalizable to patients with more comorbidities [66]. In addition, there is mixed evidence for the association between nightmares and suicide [67]. Nonetheless, further observational and genetic research to test the association between nightmares, insomnia, and SB is warranted. Moreover, although most of the used cohorts included only European ancestry, two cohorts included a minority of other ancestries: SCZ (included 20% East Asian ancestry), and SB-Cohort-2020 (included 4% African and 6% East Asian ancestry). Although only a small percentage, and accounted for in the original GWAS; this could still introduce a minor limitation in this article. Lastly, as RLS and insomnia (and psychiatric disorders) research moves from subjective symptoms to objective biomarkers to diagnose these disorders more accurately, genetic proxies utilizing these biomarker-driven GWAS might create a more homogeneous genetic signature and more precise MR results.

In conclusion, this two-sample MR analysis demonstrated for the first-time robust evidence for a potentially independent and causal effect of insomnia on SB. This finding encourages further clinical trials targeting insomnia for the prevention and treatment of SB.

## Supplementary information

Supplementary Tables

Supplementary text

Supplementary figures
