# Supplementary material for: Genetic evidence for a potential causal relationship between insomnia symptoms and suicidal behavior: a Mendelian randomization study
Source: Neuropsychopharmacology. 2022 May 10;47(9):1672–9. doi: 10.1038/s41386-022-01319-z (PMC9283512; doi:10.1038/s41386-022-01319-z)

**Supplemental Figures**

Figure 1S. Insomnia MR to MDD Leave one out egger


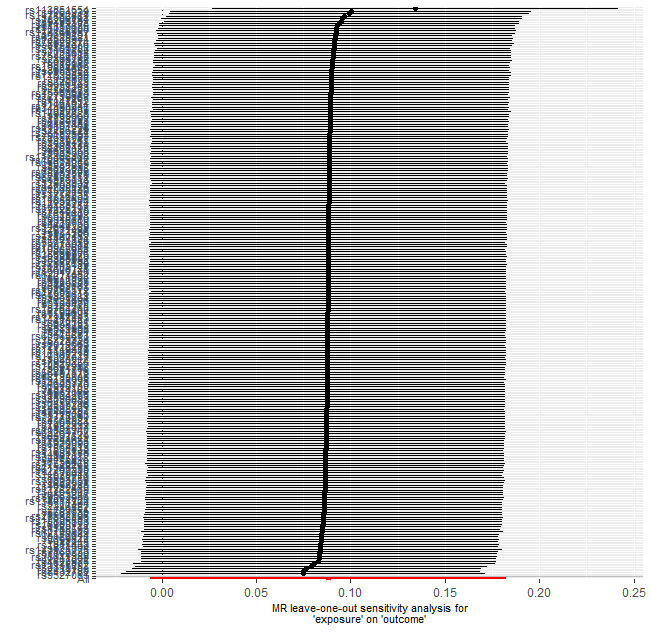


Figure 2S. Insomnia MR to BP Leave one out egger


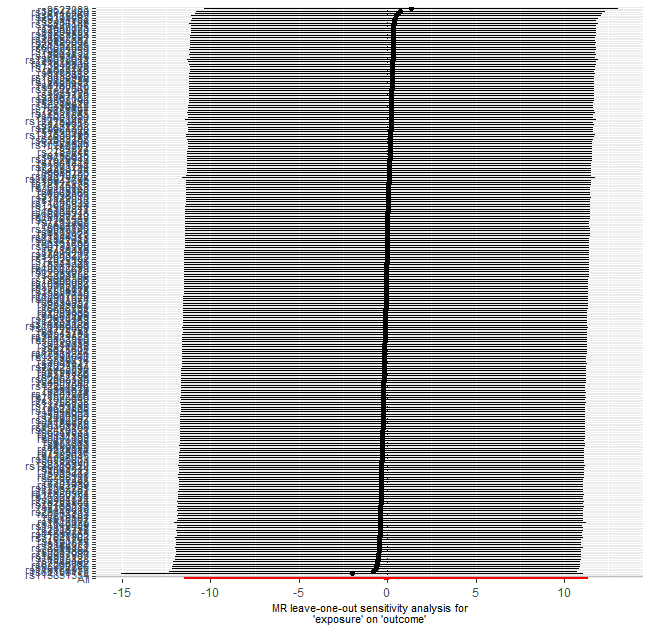


Figure 3S. Insomnia MR to SB-Cohort_2019 Leave one out egger


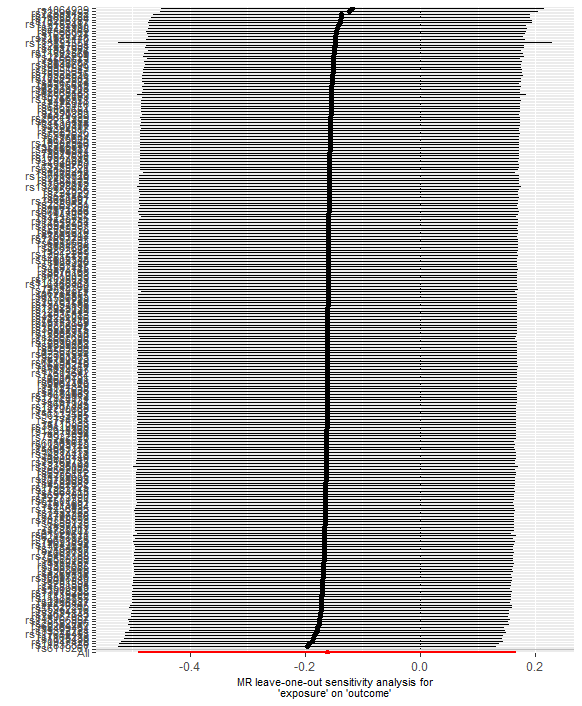


Figure 4S. Insomnia MR to SB in MDD Leave one out egger


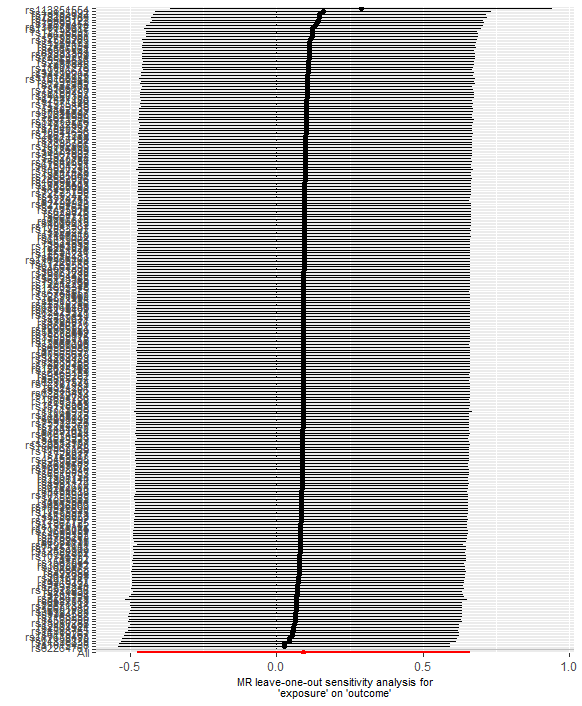


Figure 5S. Insomnia MR to SB-Cohort_2020 Leave one out egger


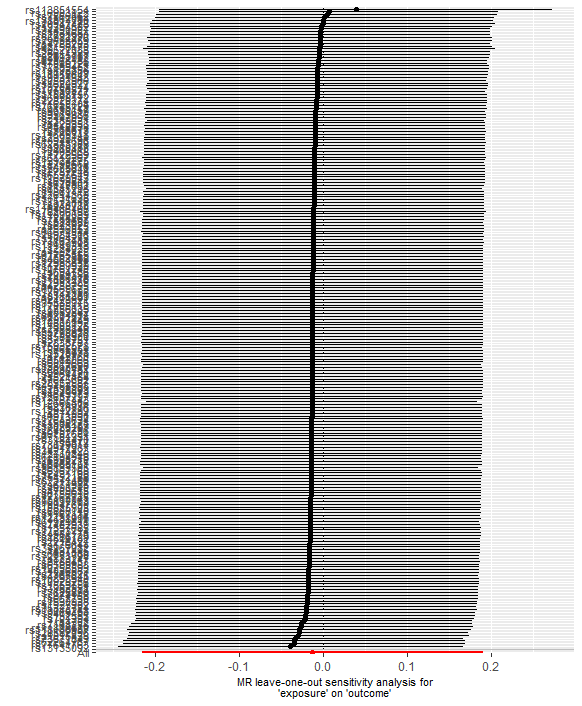


Figure 6S. Insomnia MR to MDD Leave one out IWV


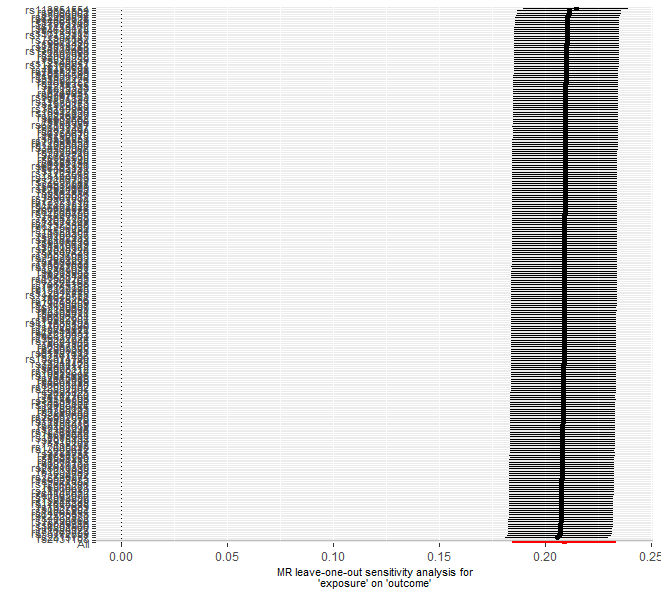


Figure 7S. Insomnia MR to BP Leave one out IWV


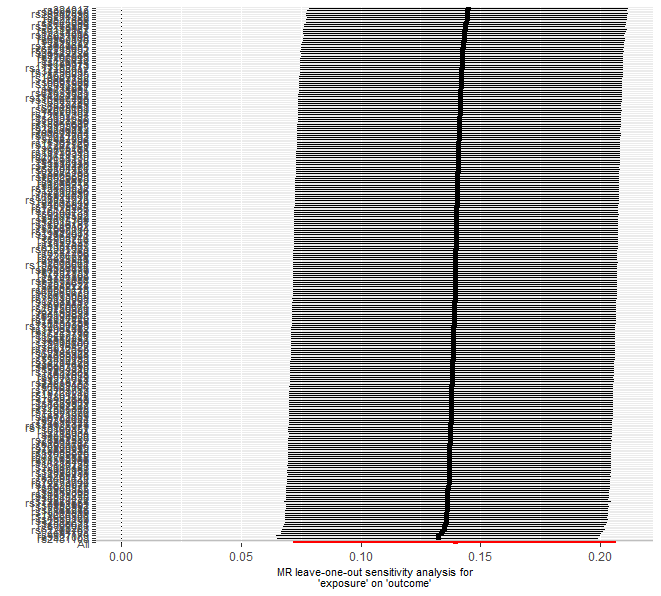


Figure 8S. Insomnia MR to SB-Cohort-2019 Leave one out IWV


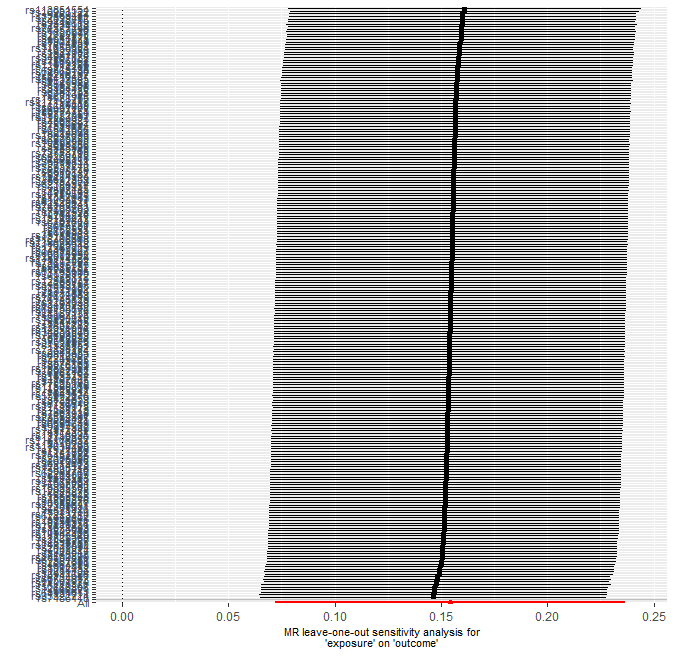


Figure 9S. Insomnia MR to SB in MDD Leave one out IWV


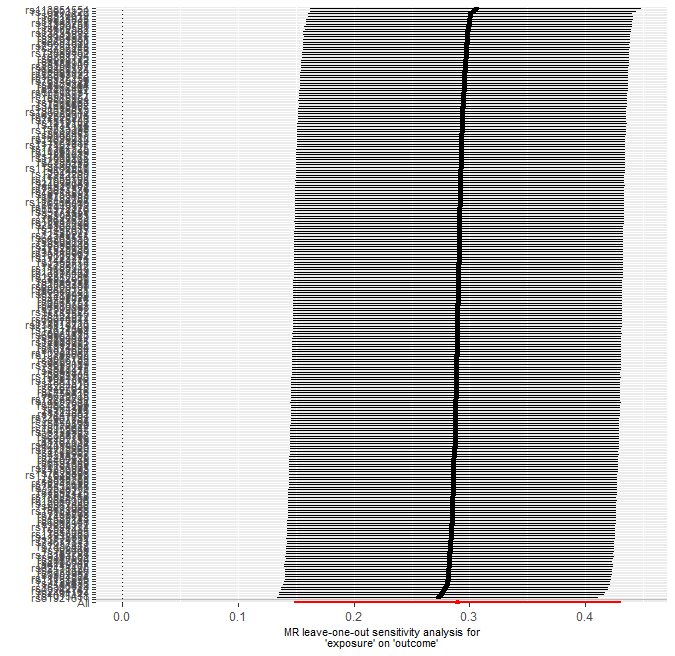


Figure 10S. Insomnia MR to SB-Cohort-2020 Leave one out IWV


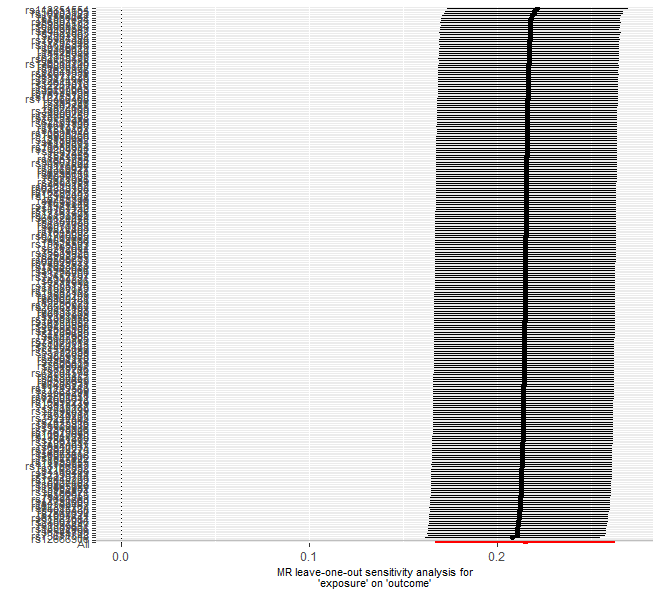

Supplement: Supplementary file 3 — Supplementary figures [file 41386_2022_1319_MOESM3_ESM.docx]
